# Health of the Army

**Published:** 1864-01

**Authors:** 


*Health of the Army.*—The following extract comprises all that is said in the report of the Secretary of War to Congress, of the Medical Department of the Army:

By the report of the Acting Surgeon-General, the department is informed that the latest reports received give one hundred and eighty-two general hospitals, with a capacity of 84,472 beds. The number of patients remaining in general hospitals June 30, 1863, was 9-1 per cent., and in the field 4-4 per cent. of the entire mean strength of the army, of whom 11 per cent. were sick, and 2-5 per cent. wounded. The corps of medical inspectors, by the system of inspections established, has added materially to the efficiency of the medical and hospital service, and a marked improvement in all matters of sanitary precaution and police is exhibited. Companies of the second battalion, invalid corps, have in many instances been advantageously substituted for contract nurses, attendants, and cooks in the general hospitals. Appropriations are asked for the payment of washing in those hospitals and on transports, where a sufficient number of matrons cannot be employed; for the collection and preservation of pathological specimens in the army medical museum; and for the preparation and examination of drugs, in connection with the purveying depots. The health of the troops has been good, and the mortality less than the preceding year.

